# Supplementary material for: Validity of the cell‐extracted proteome as a substrate pool for exploring phosphorylation motifs of kinases
Source: Genes Cells. 2023 Sep 2;28(10):727–35. doi: 10.1111/gtc.13063 (PMC11447832; doi:10.1111/gtc.13063)
Supplement: Supplementary file 1 — Figure S1. Identified phosphorylated peptides and their properties. Figure S2. Relationship between in vitro CK2 or PKA substrates obtained and protein expression levels. Figure S3. Evaluation of kinase substrate prediction performance using each cell‐line‐derived PWM. Figure S4. ROC curve analysis for prediction of kinase substrates using each cell‐derived PWM. Figure S5. The prediction performance of kinase substrates using PWM score and PL‐PWM score. [file GTC-28-727-s002.docx]

**Supplementary material**

**Validity of the cell-extracted proteome as a substrate pool for exploring phosphorylation motifs of kinases**

Tomoya Niinae^1^, Naoyuki Sugiyama^1^, Yasushi Ishihama*^1,2^

1)   Graduate School of Pharmaceutical Sciences, Kyoto University, Kyoto 606–8501, Japan

2) Laboratory of Clinical and Analytical Chemistry, National Institute of Biomedical Innovation, Health and Nutrition, Ibaraki, Osaka 567-0085, Japan.

*Correspondence and requests for materials should be addressed to Y.I. (email: yishiham@pharm.kyoto-u.ac.jp).

**Figure S1. Identified phosphorylated peptides and their properties.**

**Figure S2. Relationship between *in vitro* CK2 or PKA substrates obtained and protein expression levels.**

**Figure S3. Evaluation of kinase substrate prediction performance using each cell-line-derived PWM.**

**Figure S4. ROC curve analysis for prediction of kinase substrates using each cell-derived PWM.**

**Figure S5. The prediction performance of kinase substrates using PWM score and PL-PWM score.**

**Table S1. Peptides identified in the global proteome experiments or *in vitro* kinase reaction experiments.**

**(a) Phosphopeptides identified from *in vitro* kinase reaction using CK2 (.xlsx)**

**(b) Phosphopeptides identified from *in vitro* kinase reaction using ERK1 (.xlsx)**

**(c) Phosphopeptides identified from *in vitro* kinase reaction using PKA (.xlsx)**

**(d) Proteins identified from HeLa, MCF7 or Jurkat cells (.xlsx)**

**Table S2 *In vitro* substrates identified from *in vitro* kinase reaction experiments (.xlsx).**

**(a) *In vitro* substrates identified from in vitro kinase reaction using CK2**

**(b) *In vitro* substrates identified from in vitro kinase reaction using ERK1**

**(c) *In vitro* substrates identified from in vitro kinase reaction using PKA**

**Table S3 PWM generated from each class (.xlsx).**

**(a) PWM for CK2**

**(b) PWM for ERK1**

**(c) PWM from 400 *in vitro* substrates for PKA**

**(d) PWM from 1000 *in vitro* substrates for PKA**


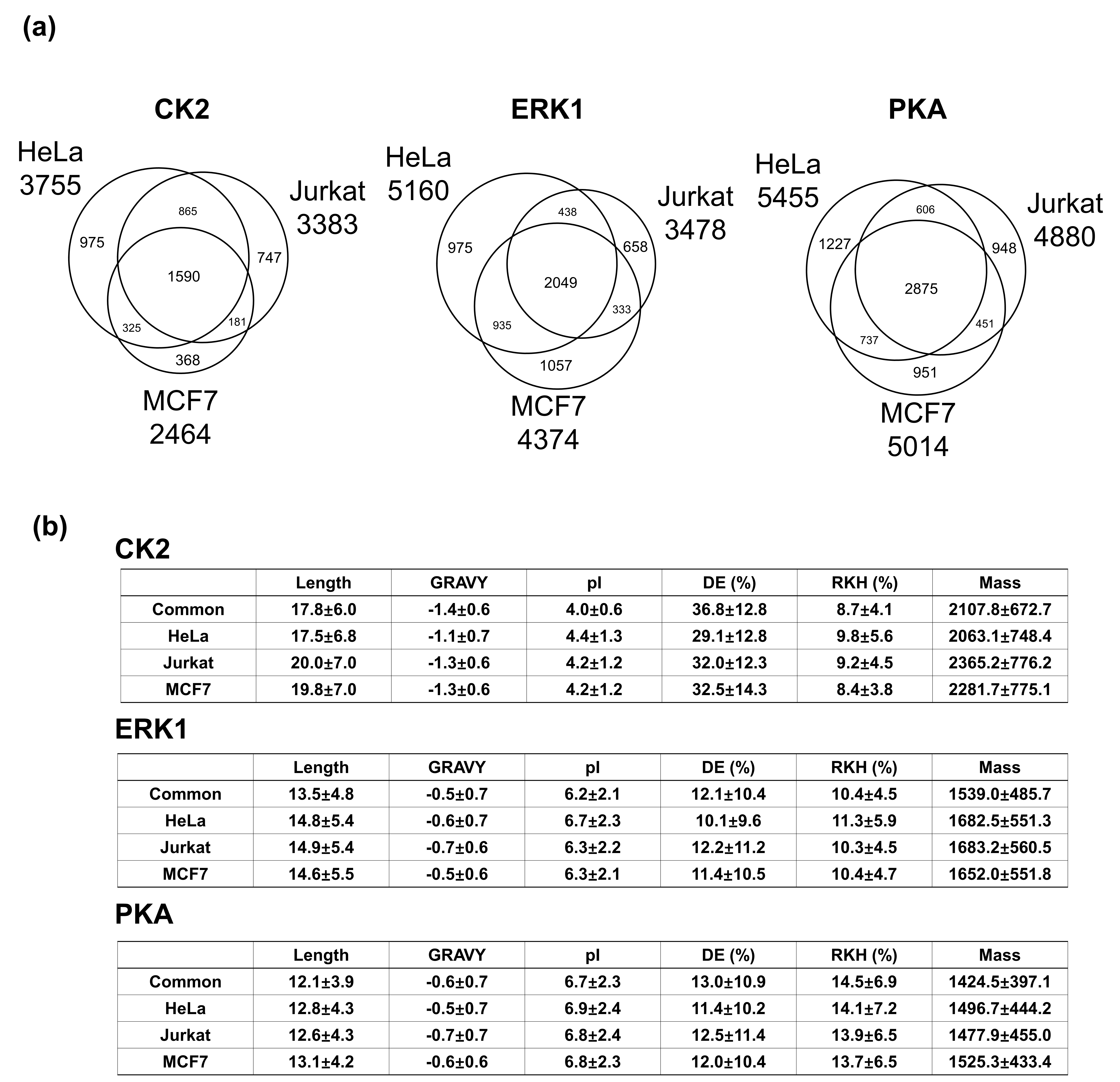


**Figure S1. Identified phosphorylated peptides and their properties.**

1. Venn diagram of phosphopeptides identified from three cell lines.

Phosphopeptides with at least one phosphorylation site with a localization probability of 0.75 or higher were counted. Phosphopeptides identified in control samples were excluded. The number of identified phosphopeptides for each cell line was counted from the total number of phosphopeptides without redundancy in triplicate.

1. Properties of identified substrate peptides from three cell lines.

Phosphopeptides commonly identified in all three cell lines are labeled Common. Phosphopeptides uniquely identified in each cell line are labeled HeLa, Jurkat or MCF7. The GRAVY score was calculated using Sequence Manipulation Suite (<https://www.bioinformatics.org/sms2/protein_gravy.html>) (Kyte & Doolittle, 1982). The pI values were calculated using Expasy Compute pI/Mw tool (<https://web.expasy.org/compute_pi/>) (Bjellqvist et al. 1993; Bjellqvist et al. 1994). The GRAVY score and the pI of the phosphorylated peptides were calculated using the unmodified sequence information. GRAVY scores and pI for phosphopeptides were calculated using unmodified sequence information.

**
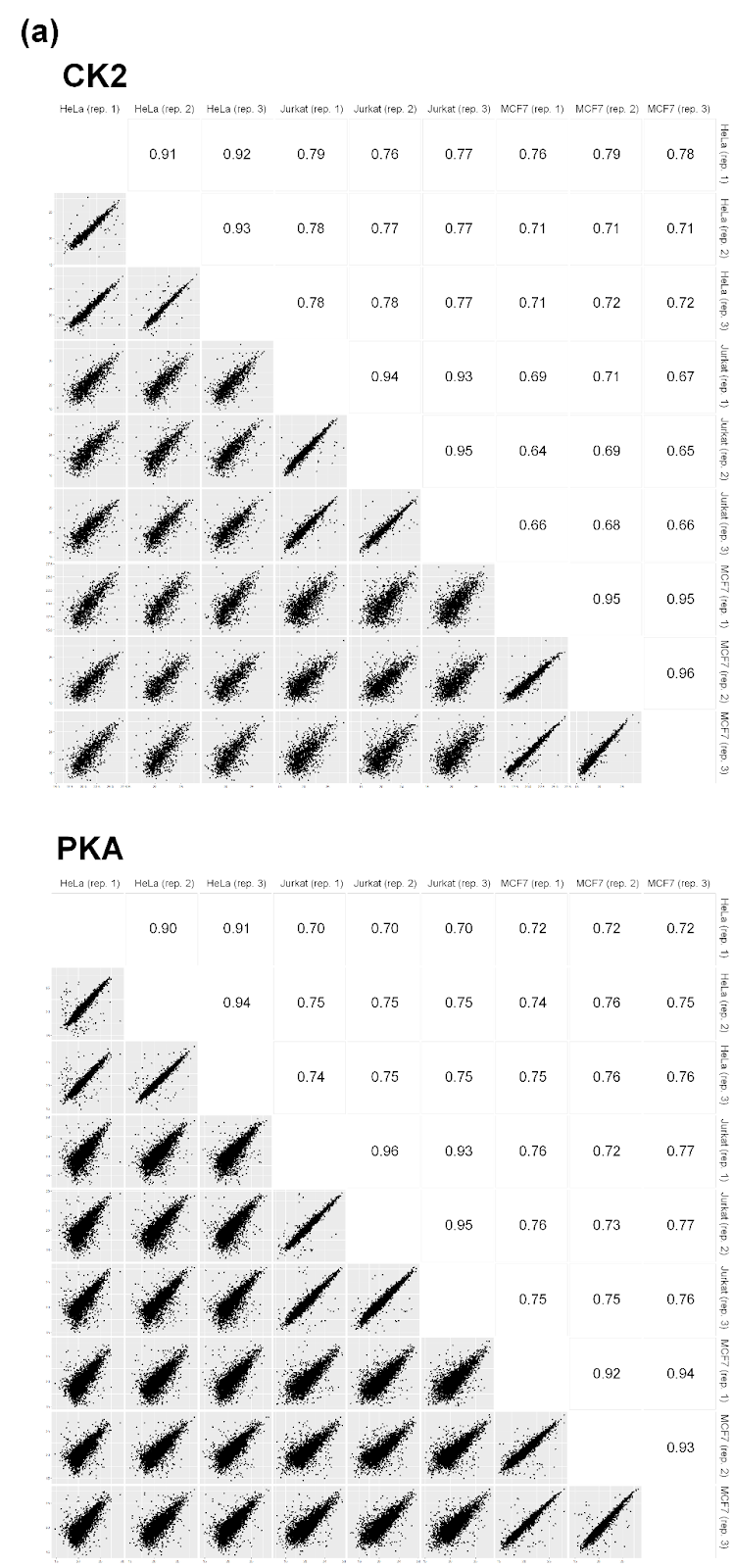
**


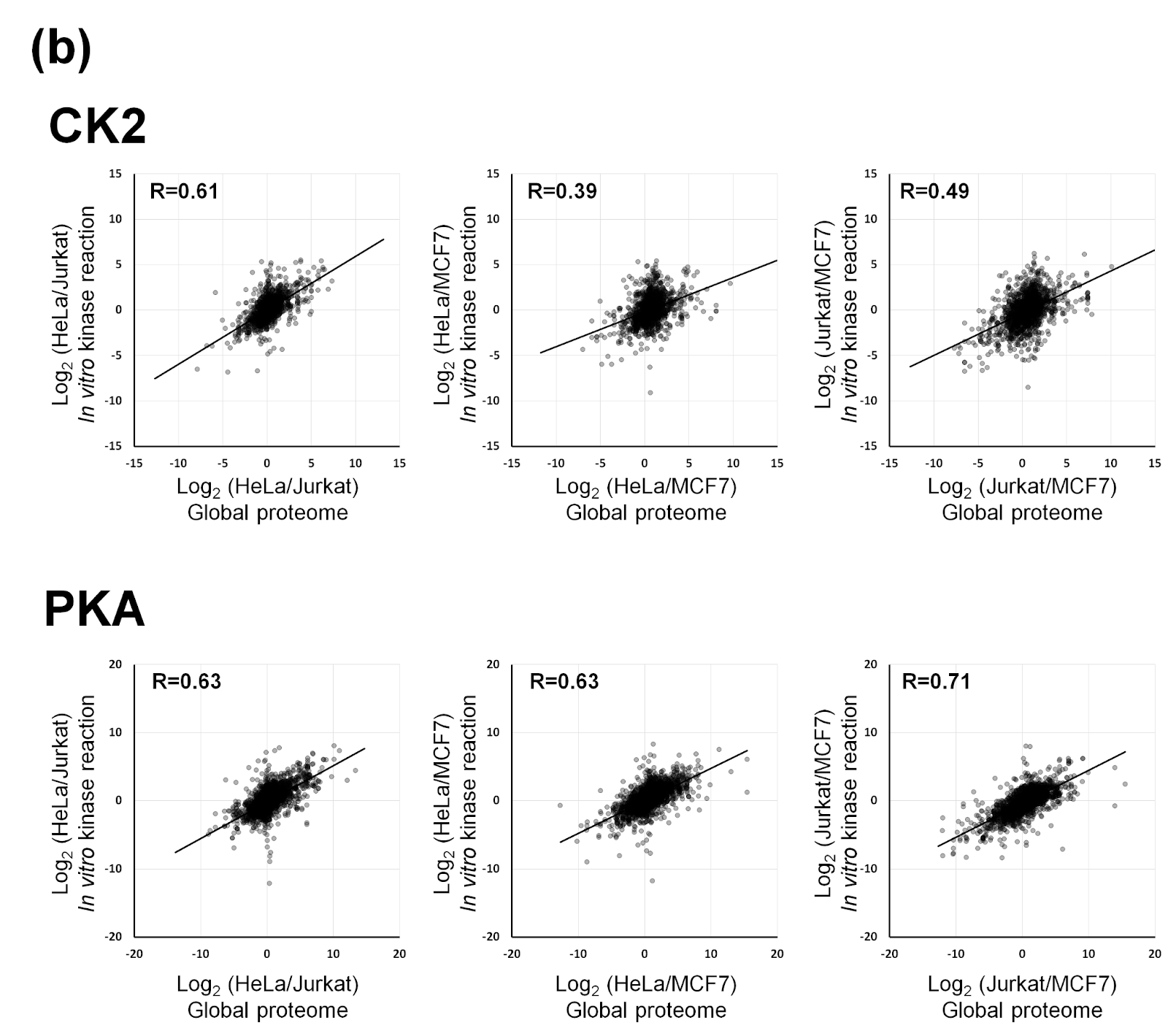


**Figure S2. Relationship between *in vitro* CK2 or PKA substrates and protein expression levels.**

1. Correlations of signal intensities of *in vitro* CK2 or PKA substrates derived from triplicate experiments with each cell line.

Pearson correlation coefficients of signal intensity (log 2) were calculated for phosphorylation sites (pS, pT) obtained by *in vitro* kinase reactions for each set of cell-extracted proteins.

1. Correlations between protein expression levels and *in vitro* CK2 or PKA substrate abundance

The normalized ratio of expression levels of a given protein between pairs of cell lines was plotted against the normalized ratio of the abundance of *in vitro* substrates on the given protein between the same pair of cell lines. The Pearson correlation coefficients are shown on the plots.


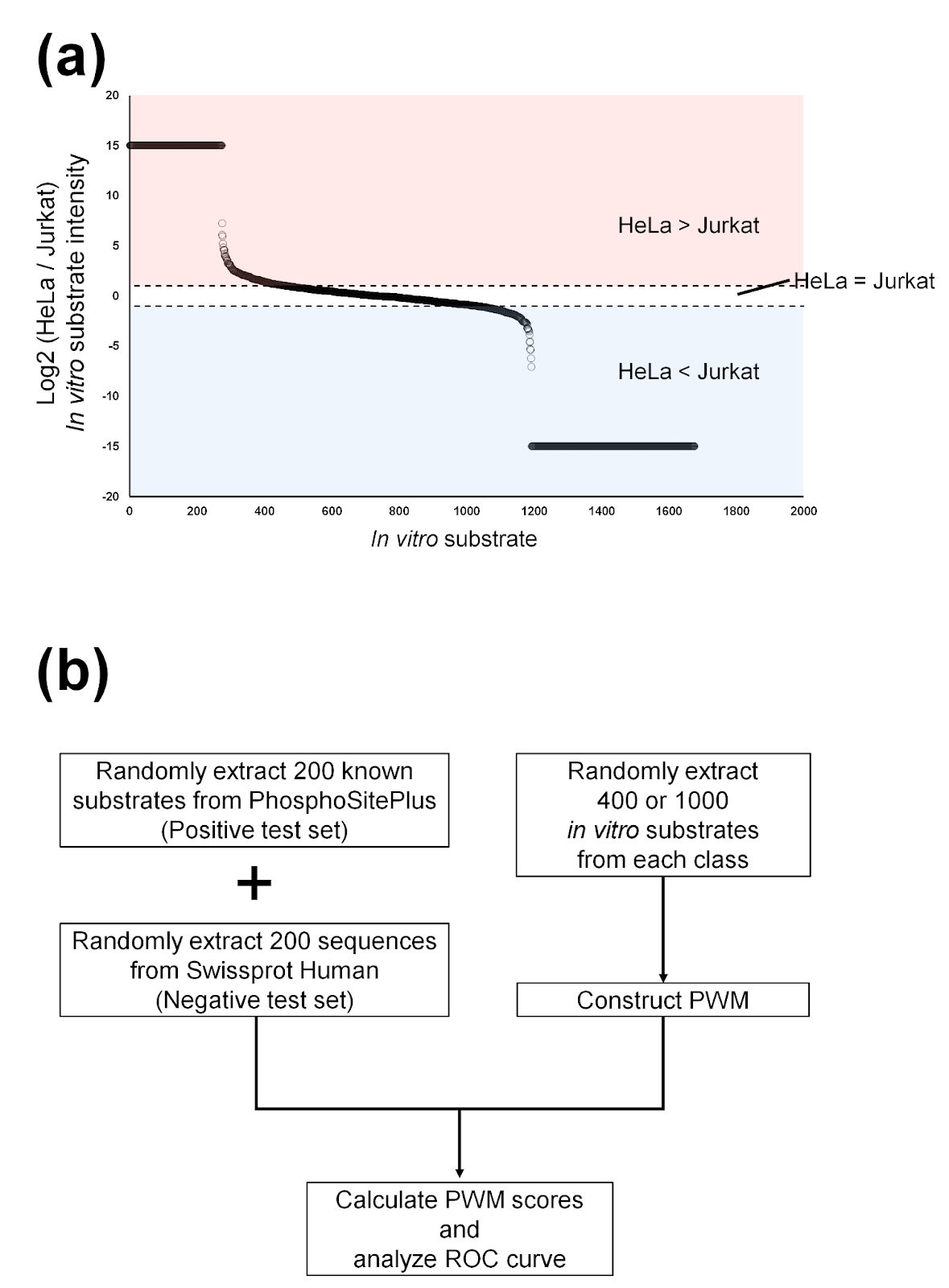


**Figure S3. Evaluation of kinase substrate prediction performance using each cell-line-derived PWM.**

(a) An example of classification based on normalized *in vitro* substrate abundance ratios.

(b) Workflow for calculation of PMW scores.


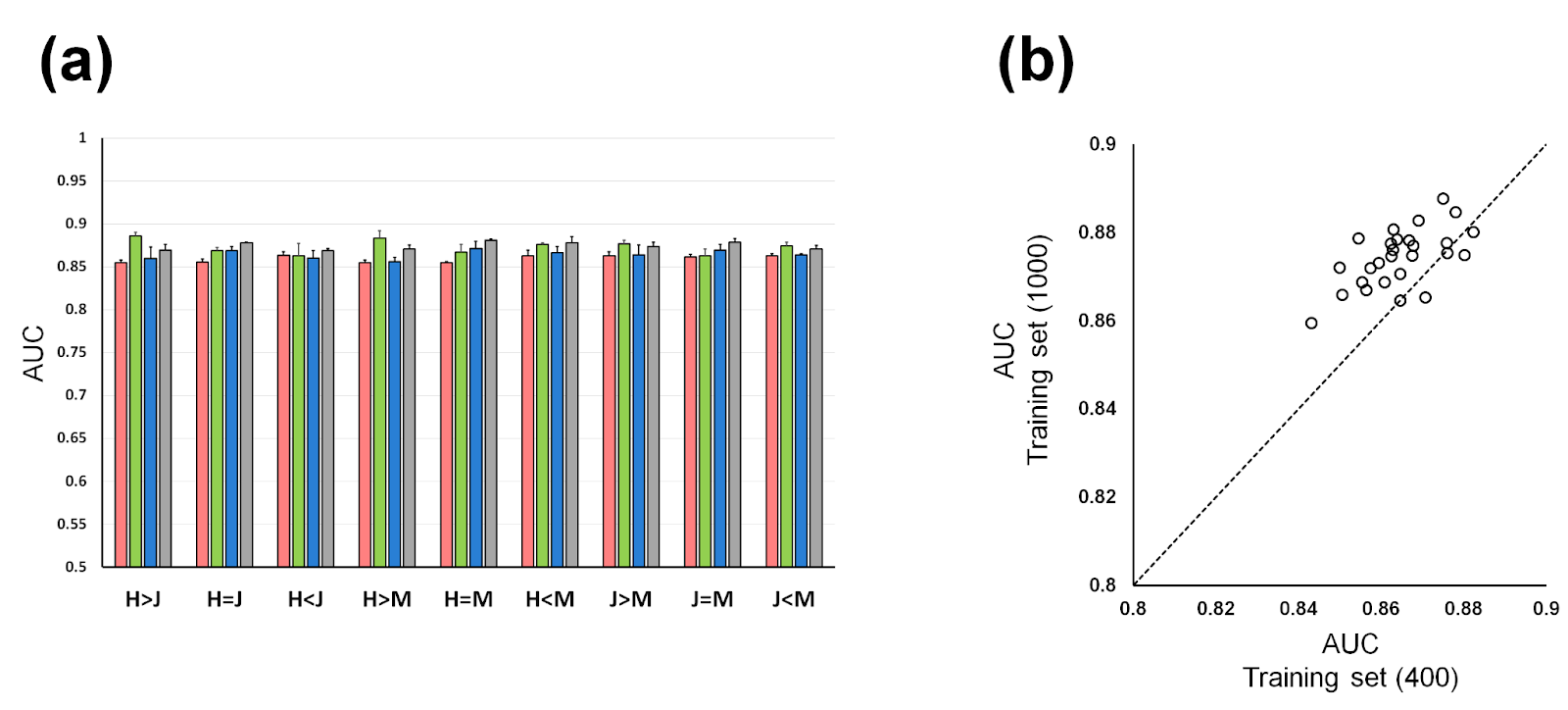


**
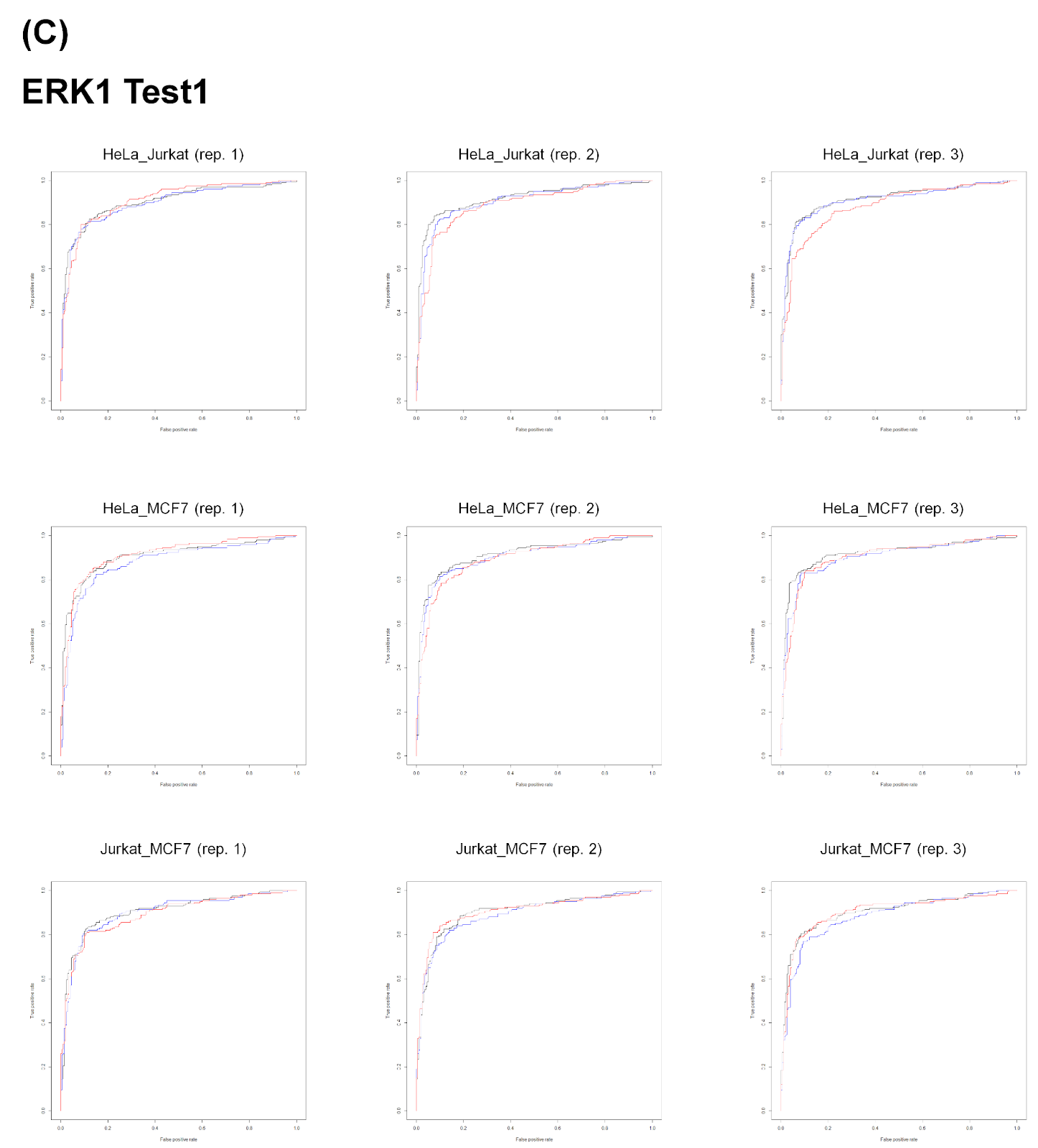
**

**
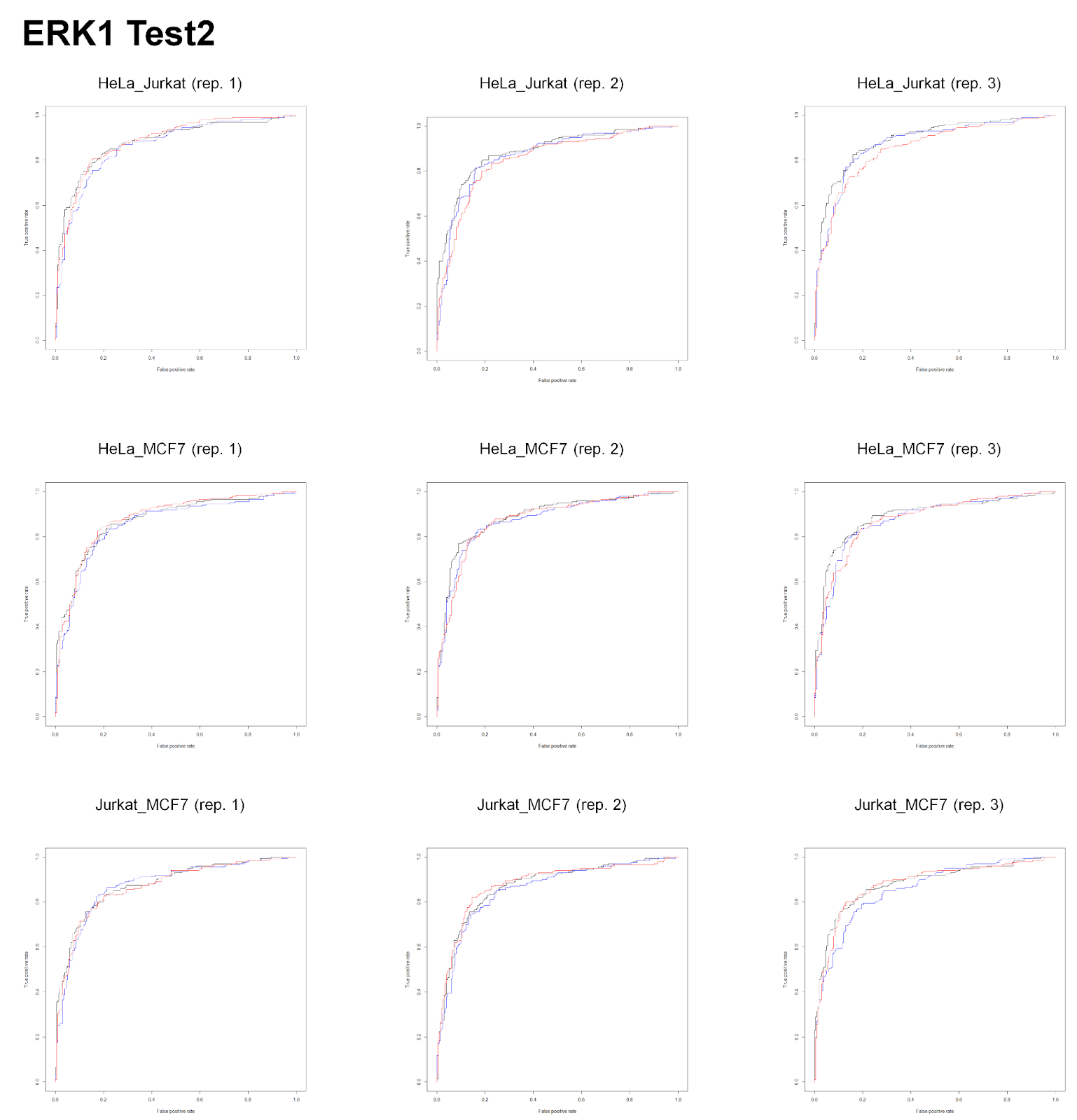
**

**
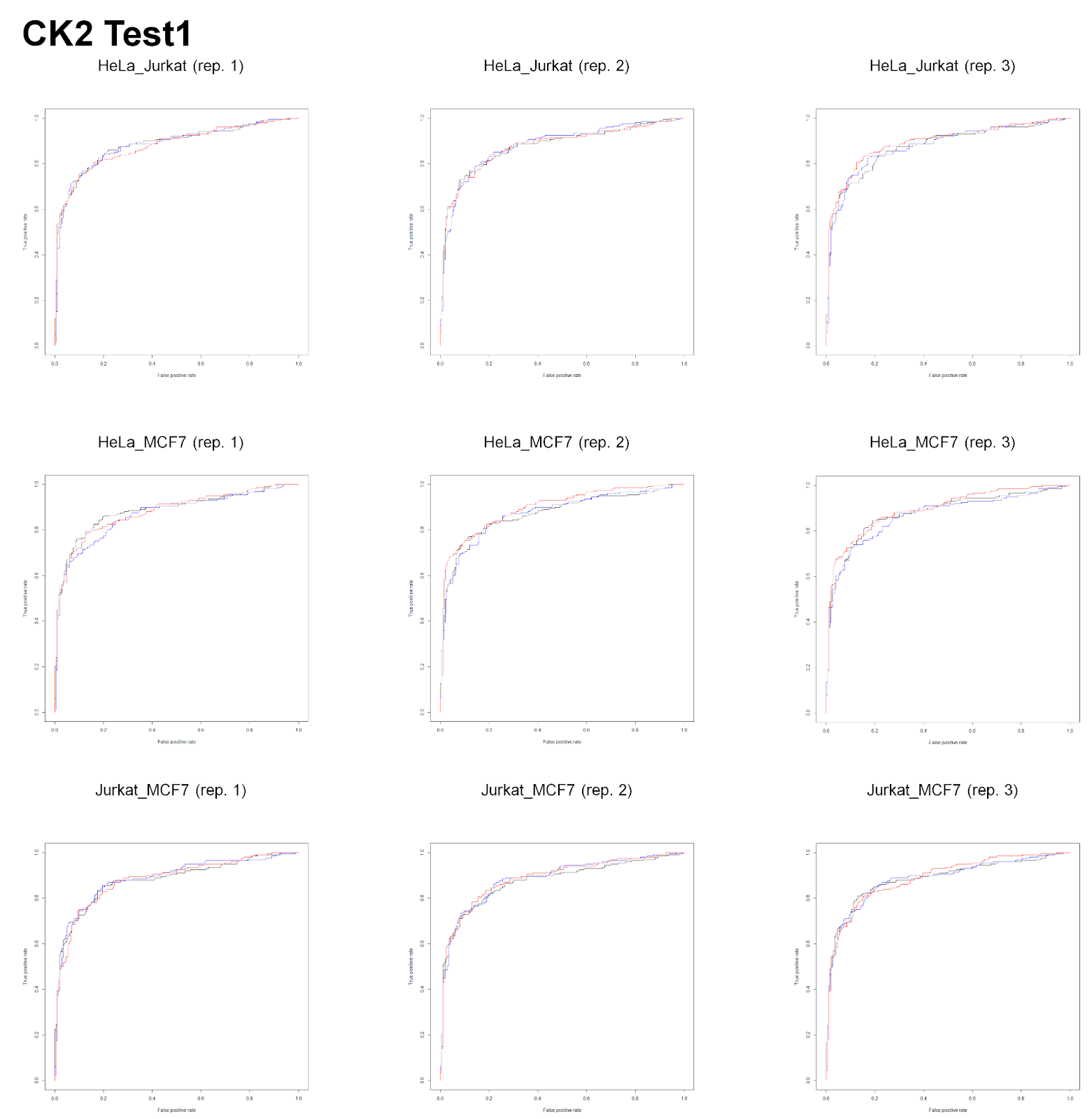
**

**
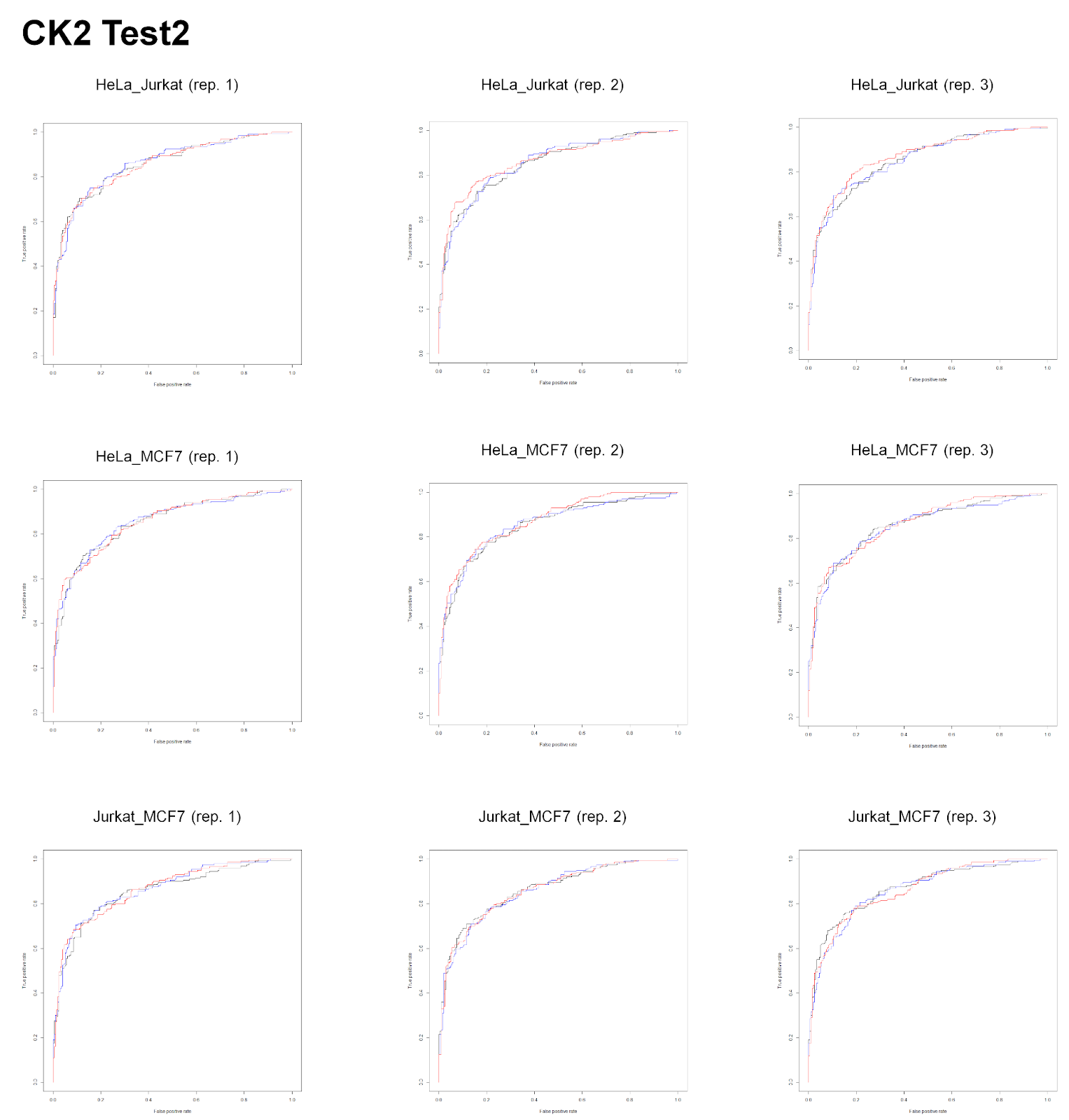
**

**
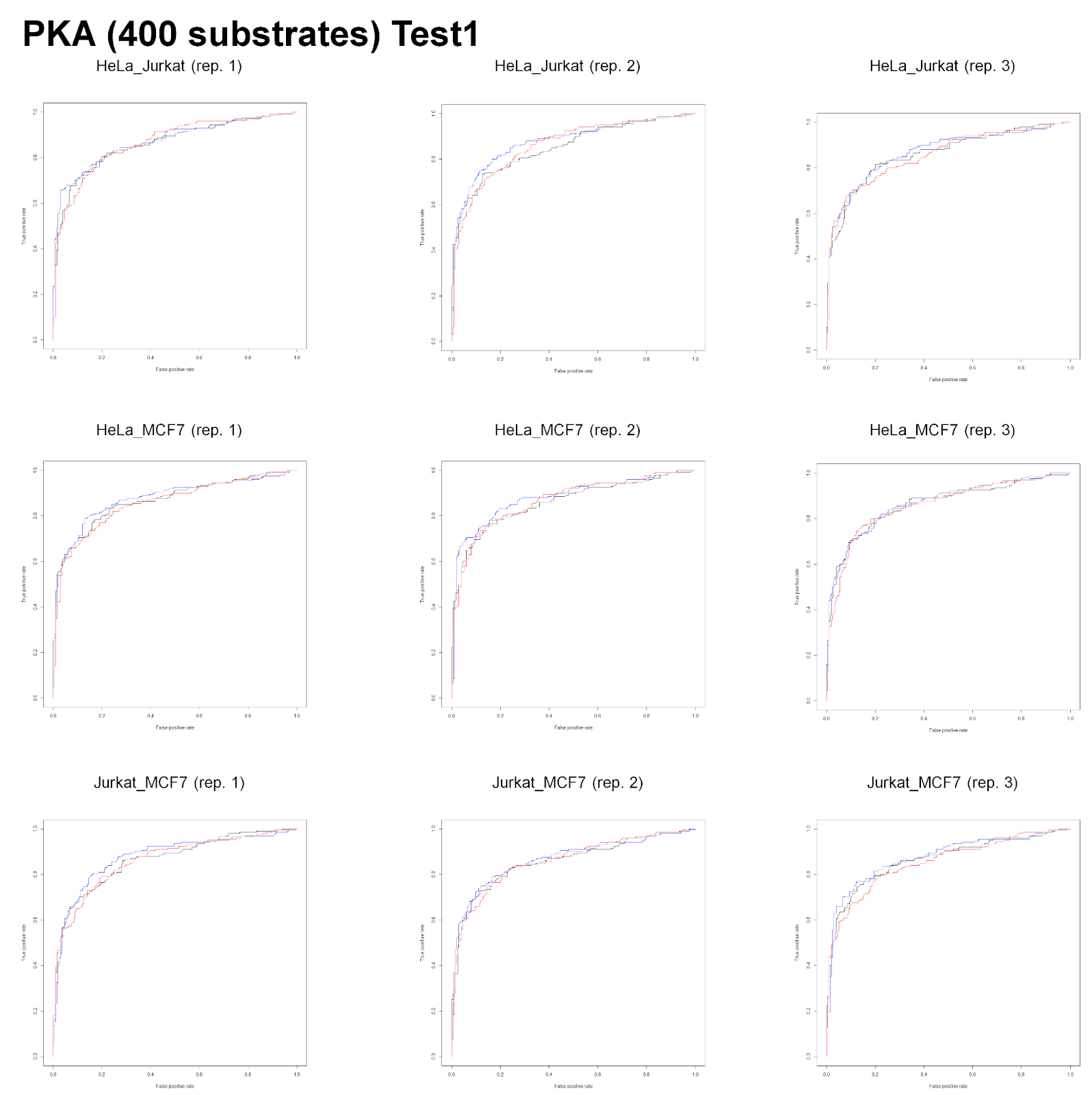
**

**
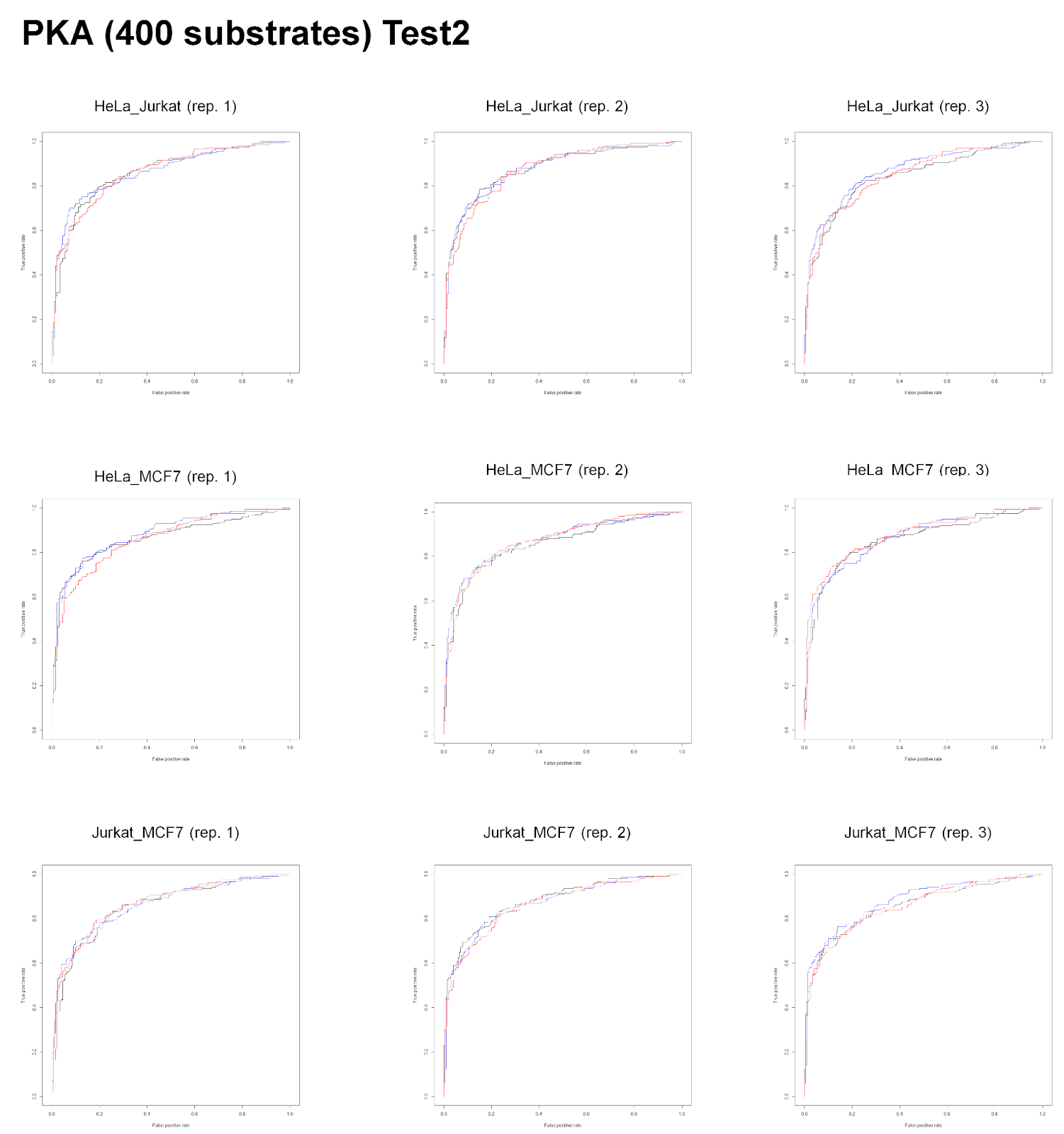
**

**
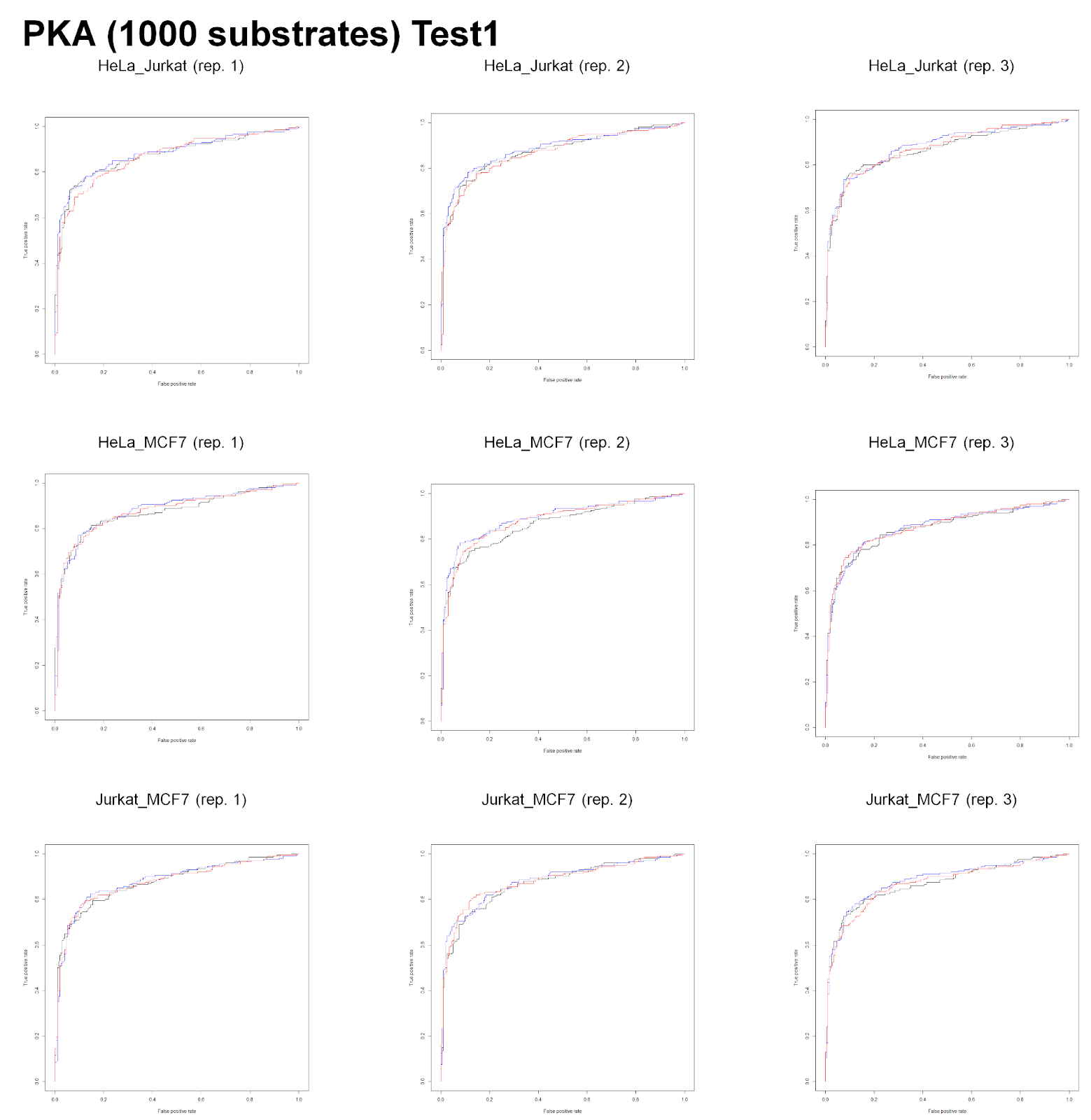
**

**
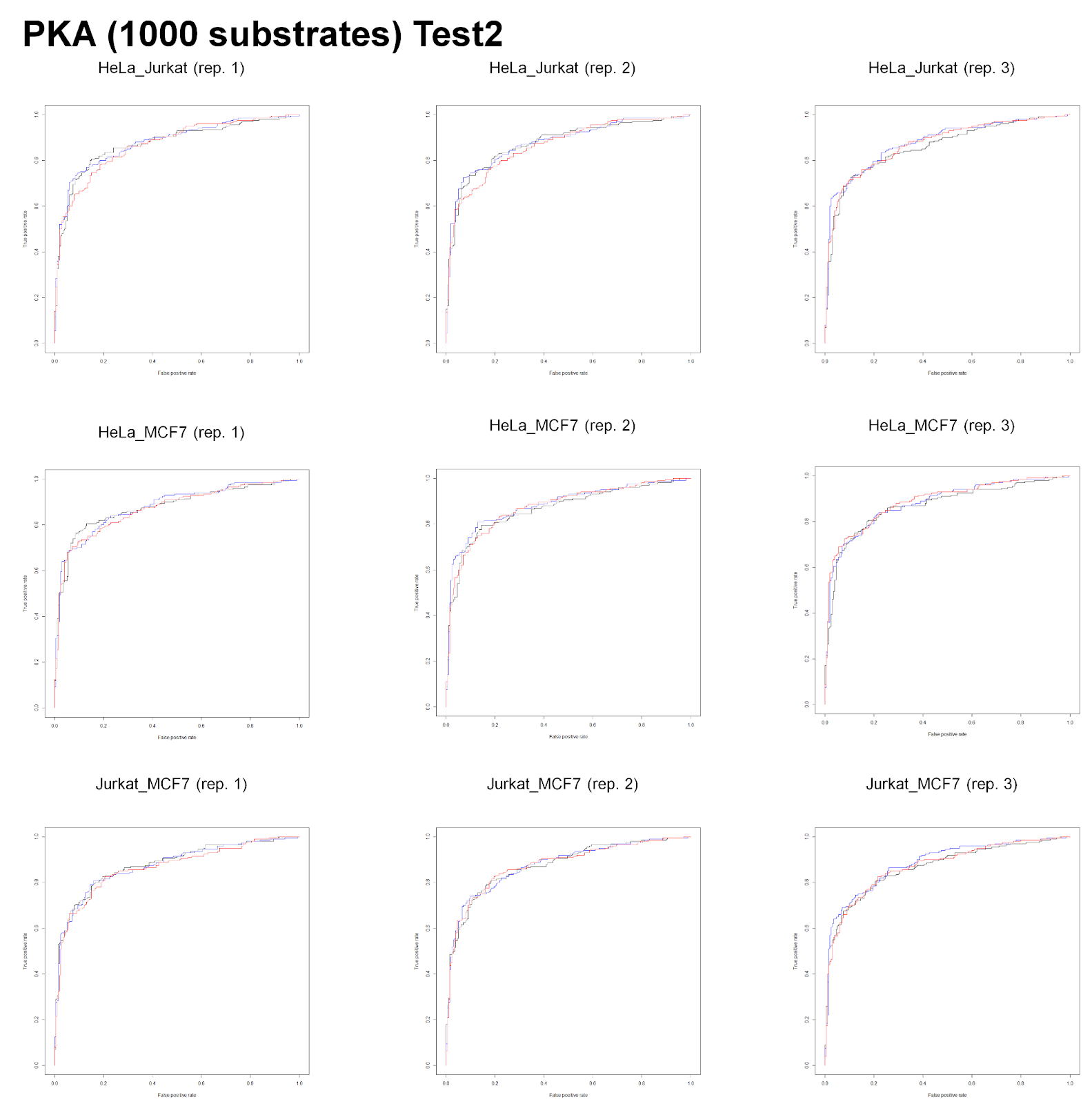
**


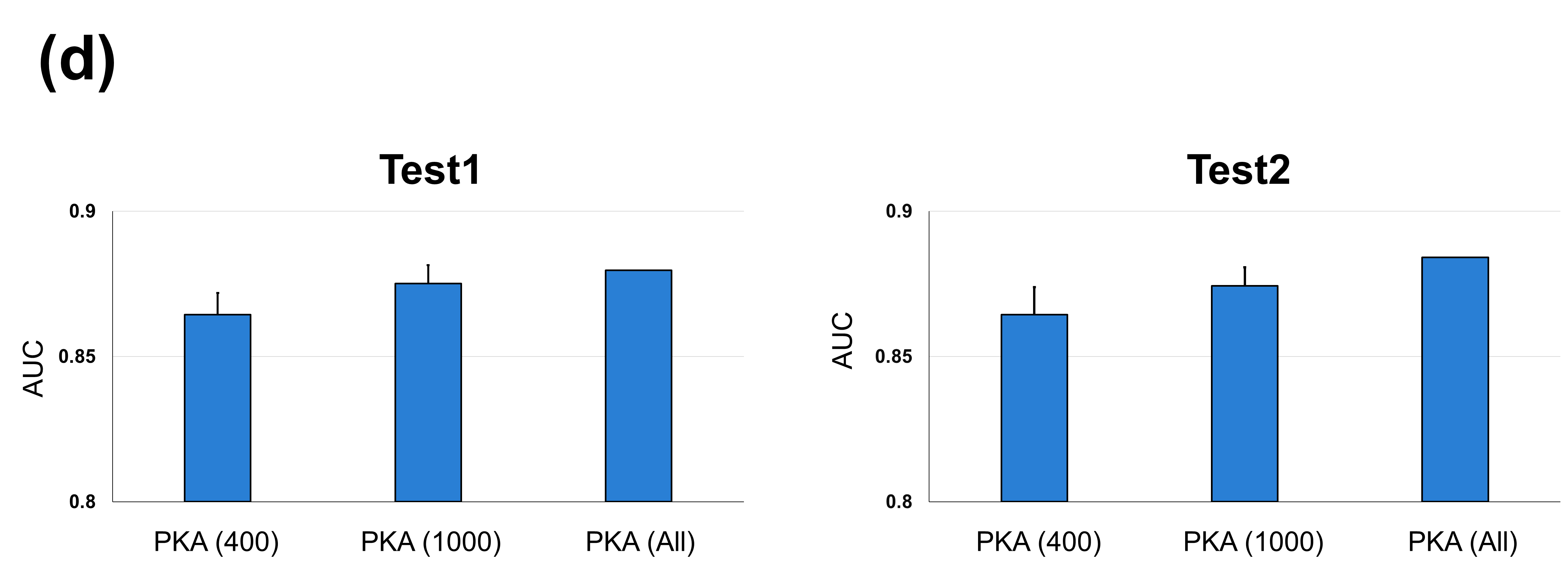


**Figure S4. ROC curve analysis for prediction of kinase substrates using each cell-derived PWM.**

(a) The kinase substrate prediction performance using each cell-derived PWM.

The kinase substrate prediction performance for each class was compared in Test 2. HeLa is indicated as H, Jurkat as J, and MCF7 as M. (Red) CK2 (Green) ERK 1 (Blue) PKA (from 400 *in vitro* substrates) (Gray) PKA (from 1000 *in vitro* substrates)

(b) Effect of *In vitro* PKA substrate number in the training set on kinase substrate predictions in Test 2.

27 training set pairs (3 classes/cell pair x 3 cell pairs x triplicates) were evaluated.

(c) ROC curve of prediction of kinase substrates with each cell-derived PWM.

(Black) H>J or H>M or J>M, (Blue) H=J or H=M or J=M, (Red) H<J or H<M or J<M

(d) Predictability of kinase substrates generated by combining PKA substrates from three cell lines.

ROC analyses using PMWs from 400 and 1000 *in vitro* substrates of each cell line are labeled PKA (400) and PKA (1000), respectively, while the analyses using PMWs from all *in vitro* substrates of all classes of all cell lines are labeled PKA (all)**.**


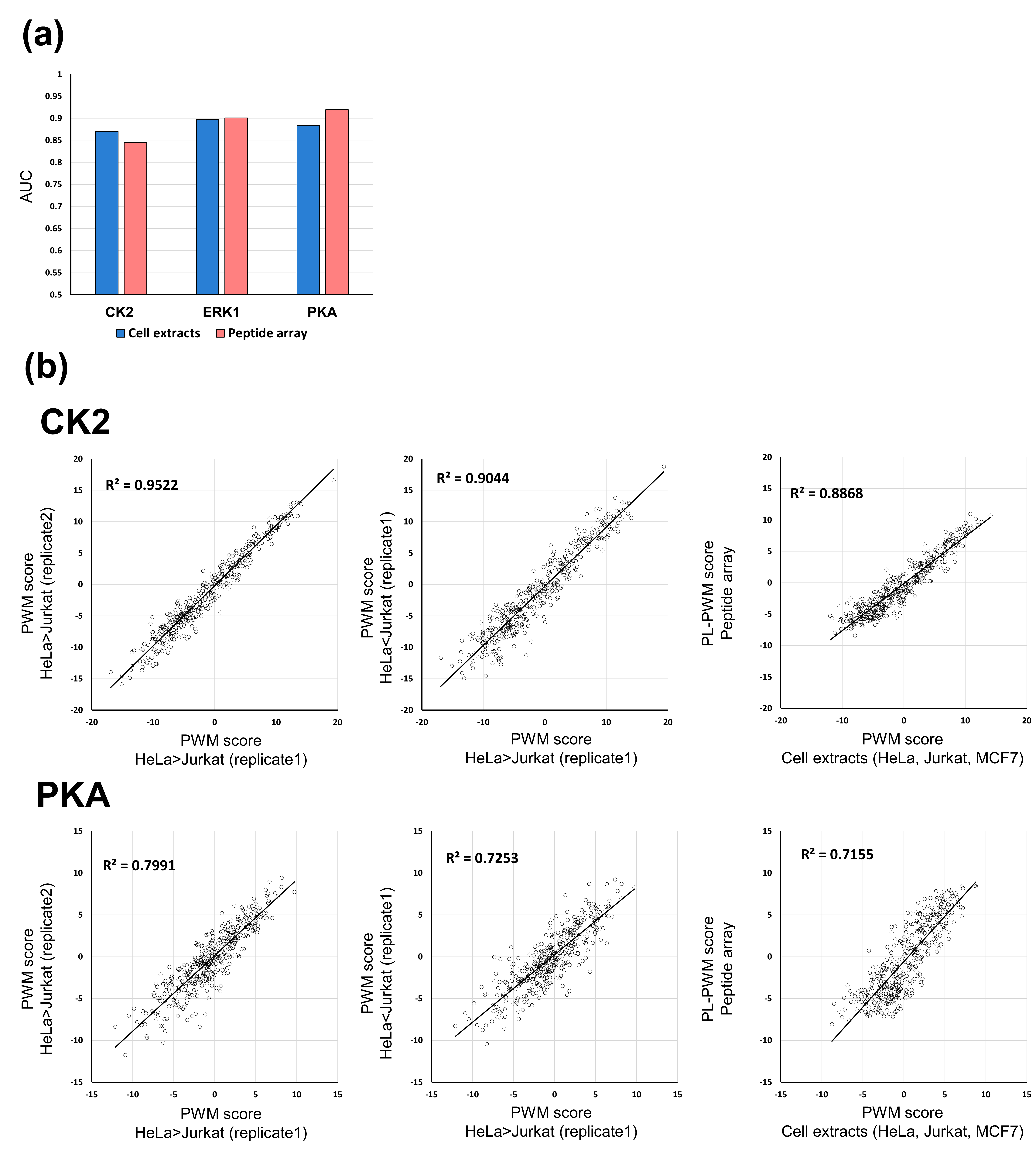


**Figure S5. The prediction performance of kinase substrates using PWM score and PL-PWM score.**

(a) ROC curve analysis for prediction of kinase substrates using PWM score and PL-PWM score.

The kinase substrate prediction performance for each class was compared in Test 2.

(b) Correlation between PWM scores and PL-PWM scores of CK2 and PKA in Test 1.

**References**

Bjellqvist, B., Hughes, G.J., Pasquali, C., Paquet, N., Ravier, F., Sanchez, J.C., Frutiger, S., & Hochstrasser, D. (1993). The focusing positions of polypeptides in immobilized pH gradients can be predicted from their amino acid sequences. *Electrophoresis* **14**, 1023–1031.

Bjellqvist, B., Basse, B., Olsen, E., & Celis, J.E. (1994). Reference points for comparisons of two-dimensional maps of proteins from different human cell types defined in a pH scale where isoelectric points correlate with polypeptide compositions. *Electrophoresis* **15**, 529–539.

Kyte, J., & Doolittle, R.F. (1982). A simple method for displaying the hydropathic character of a protein. *J. Mol. Biol.* **157**, 105–132.
